# Comparing Survival Outcomes between Hemodialysis and Hemodiafiltration Using Real-World Data from Brazil

**DOI:** 10.3390/jcm13020594

**Published:** 2024-01-19

**Authors:** Erica Pires da Rocha, Christiane Akemi Kojima, Luis Gustavo Modelli de Andrade, Daniel Monte Costa, Andrea Olivares Magalhaes, Whelington Figueiredo Rocha, Leonardo Nunes de Vasconcelos Junior, Maria Gabriela Rosa, Carolina Steller Wagner Martins

**Affiliations:** 1 NefroStar Kidney Care, Osasco 06010-067, Brazil; christiane.kojima@nefrostar.com.br (C.A.K.); daniel.monte@nefrostar.com.br (D.M.C.); andrea.olivares@nefrostar.com.br (A.O.M.); whelington@nefrostar.com.br (W.F.R.); leonardo.vasconcelos@nefrostar.com.br (L.N.d.V.J.); maria.rosa@nefrostar.com.br (M.G.R.); carolina.steller@nefrostar.com.br (C.S.W.M.); 2 Departament of Internal Medicine, UNESP, Universidade Estadual Paulista, Sao Paulo 01049-010, Brazil; gustavo.modelli@unesp.br

**Keywords:** renal dialysis, hemodiafiltration, real word data, kidney failure, chronic, treatment outcome

## Abstract

The CONVINCE trial demonstrates that high-dose hemodiafiltration offers a survival advantage for patients in the high-flux hemodiafiltration group compared to hemodialysis. We compared the outcomes of hemodialysis and hemodiafiltration using real-world data. We conducted an analysis on a cohort of patients who underwent hemodiafiltration therapy (HDF) at a single center, NefroStar Clinics. The results obtained were then compared with data from patients receiving hemodialysis (HD) therapy within the Brazilian Public Health System (SUS). The primary outcome was mortality from any cause. Results: A total of 85 patients undergoing hemodiafiltration were compared with 149,372 patients receiving hemodialysis through the Brazilian Public Health System (SUS). Using a 2:1 propensity score, we compared the 170 best-match HD patients with 85 HDF patients. In the Cox analysis, HDF therapy showed a reduced risk of mortality with an HR of 0.29 [0.11–0.77]. The propensity score analysis showed a HR of 0.32 [95% CI: 0.11–0.91]. This analysis was adjusted for age, type of access, KT/v, hemoglobin, and phosphorus. The Kaplan–Meier analysis showed respective survival rates for HDF and HD at the end of one year, 92.1% and 79.9%, *p* < 0.001. These results suggest high-flux hemodiafiltration has survival advantages over hemodialysis in a real-world scenario.

## 1. Introduction

Chronic kidney disease (CKD) is a globally prevalent and debilitating condition that significantly impacts the quality of life and overall health [1]. As renal function declines, the accumulation of toxins, electrolyte imbalances, and fluid overload imposes the implementation of renal replacement therapies (RRT) to maintain patient physiological equilibrium. Among the most common RRT options, hemodialysis (HD) and hemodiafiltration (HDF) have emerged as important modalities for managing end-stage renal disease (ESRD) [2].

Hemodialysis, a cornerstone of renal replacement therapy for decades, involves the extracorporeal circulation of blood through a dialyzer, where toxins and fluids are diffused across a semipermeable membrane into a dialysate solution [3]. Although HD has demonstrated its efficacy in the treatment of CKD patients, concerns persist about its limited capacity to adequately clear larger molecular-weight solutes, such as middle molecules and cytokines, which have been implicated in several complications associated with CKD [4,5].

Hemodiafiltration, a relatively newer modality, offers an innovative approach to renal replacement therapy by combining the principles of hemodialysis and convective solute transport [6]. This technique integrates diffusive and convective clearance mechanisms, allowing for the efficient removal of a broader spectrum of solutes, including those of higher molecular weight [7]. The concomitant use of diffusion and convection in HDF holds promise in addressing the inadequacies of HD, potentially leading to improved clinical outcomes, reduced morbidity, and enhanced quality of life for CKD patients [8].

Recently, the CONVINCE trial demonstrated that high-dose hemodiafiltration offers a survival advantage for patients in the high-flux hemodiafiltration group compared to hemodialysis. Among 1360 participants across 8 European countries, those receiving high-dose hemodiafiltration experienced a lower risk of all-cause mortality during a 30-month follow-up (17.3% vs. 21.95%). Notably, the survival benefit was particularly evident in patients without a history of cardiovascular disease or diabetes mellitus [9].

Although randomized controlled trials are gold standard studies to address the efficacy of a medication, or treatment/therapy, phase IV studies are necessary to validate and replicate the results in a real-world scenario [10].

The primary aim is to compare the survival advantage of high-flux hemodiafiltration with conventional hemodialysis in a real-world scenario in Brazil.

## 2. Materials and Methods

This study conducted an analysis on a cohort of patients receiving hemodiafiltration therapy at a single center, NefroStar clinics, ranging from January 2020 to June 2023. The obtained results were compared with data from patients undergoing hemodialysis therapy at the Public Health System (SUS) in Brazil during the same period. This study was approved by the Research Ethics Committee of UNESP Botucatu/SP (approval number 74334123.3.0000.5411). Informed consent was obtained from participants prior to their participation in the study. All methods were carried out in accordance with relevant guidelines and regulations.

### 2.1. Hemodiafiltration Cohort

All adult patients (>18 years) who were treated exclusively with hemodiafiltration therapy in NefroStar were included (incident and prevalent patients). We excluded patients who were treated with other modalities of renal replacement therapy and interim hemodiafiltration patients, defined as individuals who are temporarily receiving hemodiafiltration treatment at a healthcare facility other than their primary HDF center.

The hemodiafiltration protocol was designed to achieve a convective volume in post-dilution HDF greater than or equal to 69 L per week and in pre-dilution more than or equal to 120 L per week. We prefer the use of the replacement model in post-dilution HDF, but the pre-dilution modality was recommended in the following situations: when the desired convective volume is not achieved in post-dilution HDF (vascular access with low blood flow); high risk of hemoconcentration (elevated hematocrit, high concentrations of serum proteins, high blood viscosity); when there is hemodynamic instability, even in post-dilution HDF, with difficulty tolerating ultrafiltration volume (e.g., severe cardiac patients).

### 2.2. Hemodialysis Cohort

We retrieved data from patients who were treated with hemodialysis in the public health system in Brazil. The information was extracted from the outpatient information system (SIA) database. We included all consecutive adult (>18 yrs) patients who were performing hemodialysis in the same period. We excluded patients in other modalities of renal replacement therapy (peritoneal dialysis) and excluded patients with less than 3 months of therapy. The DATASUS database is anonymized and publicly accessible.

### 2.3. Collect Data

This study involved the collection of baseline information, including age, gender, and underlying diseases. Details pertaining to the dialysis access method, such as the central venous catheter and arteriovenous fistula, were also recorded. We retrieved the number of dialysis sessions conducted per week. Adequacy assessments were conducted through monthly biochemical exams. The average of monthly biochemical measurements was utilized for analysis. We assessed repeated measures of serum hemoglobin, serum phosphorus, serum parathormone (PTH), and serum albumin. The levels of hemoglobin and parathormone were categorized based on KDOQI target levels. The percentage of patients with hemoglobin levels below 10 g/dL and patients with parathormone levels above 600 ng/mL was presented. We evaluated the single-pool Kt/V (spKt/V) to assess the adequacy of hemodialysis.

### 2.4. Data Extraction from DATASUS (Hemodialysis Cohort)

The database of hemodialysis patients from the public health system in Brazil was downloaded using the microdatasus package in R [11]. The information system retrieved was SIA-SUS, which refers to the reimbursement system and is available monthly in the public health system. DATASUS is the Department of Informatics of the Unified Health System in Brazil. It is responsible for collecting, processing, analyzing, and disseminating health-related information in the country. DATASUS plays a crucial role in managing health data and supporting decision-making processes within the Brazilian health system. The SIA-SUS is one of the subsystems within DATASUS. It focuses on outpatient and ambulatory care information, particularly related to the reimbursement system. This subsystem provides data on procedures, services, and costs incurred within the public health system [11].

### 2.5. Outcome

The primary outcome of this study was mortality from any cause.

### 2.6. Statistics

Categorical data were described in absolute numbers and percentages. Numeric data were described as median, 25th percentile, and 75th percentile. For survival analysis, Kaplan–Meier curves with log-rank tests were used. Multivariate Cox regression was fitted to compare the hemodialysis and hemodiafiltration groups, using previously known risk factors that affect mortality as predictors. We used robust variance to estimate standard errors. We used survival-adjusted curves to plot survival differences between groups. The method of adjusted survival curves is a statistical approach used to create expected survival curves for different subpopulations based on a Cox proportional hazards model. The objective of this method is to provide a way to visualize and compare survival curves while accounting for the influence of various covariates or factors [12].

Sensitivity Analysis: We conducted a sensitivity analysis to evaluate the resilience of Cox regression mortality results against potential unmeasured confounding, employing the E-value methodology developed by VanderWeele and Ding [13]. This method estimates the minimum relative risk that an unmeasured confounder would need to have to nullify the observed association of reduced mortality risk in the HDF group in this study.

We conducted another sensitive analysis employing propensity scores to investigate the impact of hemodiafiltration treatment while accounting for potential confounding variables. Propensity score matching was employed to estimate the treatment effect, incorporating covariates to ensure a balanced comparison. The matching process included variables associated with the outcome based on previous reports, namely age, gender, type of vascular access, serum hemoglobin, serum phosphorus, parathormone, and serum albumin. Optimal matching on the propensity score was applied. We implemented a 2:1 ratio for matching, signifying that two control units were matched for each treatment unit. The propensity score was determined through logistic regression, modeling the treatment in relation to the covariates. Post-matching, all standardized mean differences for covariates were found to be below 0.1, indicating satisfactory balance. To ascertain the treatment effect and its standard error, a Cox model was employed, integrating matching weights in the estimation. The coefficient associated with the treatment (Hazard Ratio, HR) was considered the estimate of the treatment effect.

The R software version 4.1.2 and packages survival, survminer, and MatchIt were used for these analyses.

## 3. Results

**Figure 1:**
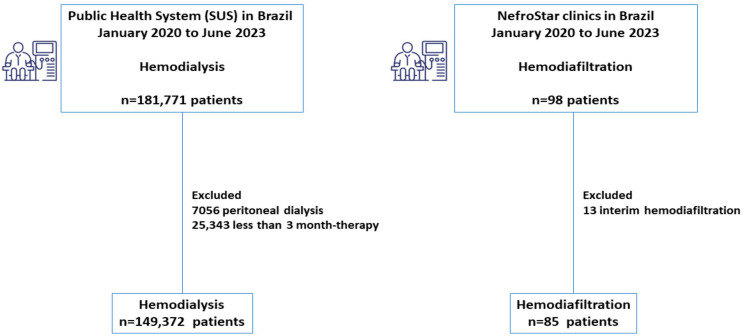
Flowchart illustrating the survival analysis of hemodiafiltration compared to the hemodialysis cohort in Brazil.

In this study, we evaluated 98 patients who underwent hemodiafiltration and 181,771 who underwent hemodialysis during the same period. In the hemodiafiltration cohort, we excluded 13 patients who had performed HDF previously in another center (interim hemodiafiltration), resulting in 85 patients. In the hemodialysis cohort, we excluded 7056 patients on peritoneal dialysis and 25,343 with less than 3 months of therapy, resulting in 149,372 patients (Figure 1).

In the hemodiafiltration cohort, the baseline kidney diseases included diabetes (25%), glomerulonephritis (13%), hypertension (16%), unknown causes (12%), and other conditions (34%). The most common comorbidities were hypertension (87%), diabetes (33%), and dyslipidemia (34%). The most frequently performed number of weekly sessions was 3 (44%), with a range of 2 to 6 sessions (2 sections, 3.5%, 4 sections, 5.9%, 5 sections, 28%, and 6 sections, 19%). The mean convective volume was 67 ± 32 L per week. Among patients who underwent three sessions per week, the median convective volume was 24 ± 10 L per session. The post-dilution convective modality was chosen in 82 (96%) patients.

In the hemodialysis cohort, the majority of patients underwent three weekly sessions (82.9%). The median age was 54 (41–67) years in the hemodiafiltration group and 56 (46–68) years in the hemodialysis group, with a *p*-value of 0.054. The percentage of arteriovenous fistulas was 61% in the hemodiafiltration group and 63% in the hemodialysis group, with no significant difference (*p* = 0.8). The single-pool Kt/v value was similar between the two groups. Hemoglobin levels were higher in the hemodiafiltration cohort, with a median of 11.44 (11.02–11.97), compared to the hemodialysis cohort, with a median of 10.25 (8.97–11.42), and the difference was statistically significant (*p* < 0.001). A higher proportion of hemodialysis patients (41%) exhibited hemoglobin levels below 10 g/dL compared to the hemodiafiltration cohort (8.5%), *p* < 0.001. Parathormone levels were lower in the hemodiafiltration cohort, with a median of 236 (142–376) pg/mL, compared to the hemodialysis cohort, with a median of 327 (166–612) pg/mL, and this difference was also statistically significant (*p* < 0.001). A higher number of patients in the hemodialysis group (26%) had parathormone levels above 600 ng/dL compared to the hemodiafiltration cohort (8.5%), *p* < 0.001.

The 12-month mortality rate was 5.9% in the hemodiafiltration group and 14% in the hemodialysis group, with a *p*-value of 0.036 (Table 1). The follow-up duration for the hemodiafiltration and hemodialysis cohorts was 391 (168–592) days and 402 (101–920) days, respectively, with a *p*-value of 0.15.

**Figure 2:**
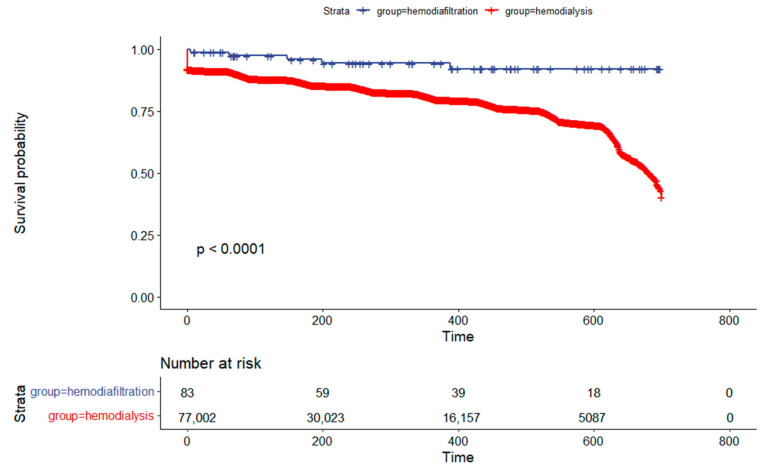
Kaplan–Meier Survival Analysis Comparing Hemodiafiltration to the Hemodialysis Cohort in Brazil.

The univariate Kaplan–Meier analysis showed survival rates for hemodiafiltration and hemodialysis at 180 days of 95.5% and 85.8%, respectively. At the end of one year, the survival rates were 92.1% for hemodiafiltration and 79.9% for hemodialysis, with a significant difference (*p* < 0.001) as illustrated in Figure 2.

In the multivariate Cox regression analysis, it was found that having an arteriovenous fistula was associated with a lower risk of death, with a hazard ratio (HR) of 0.38 (95% CI 0.35–0.41), and this association was statistically significant (*p* < 0.001). Higher levels of hemoglobin and greater single-pool Kt/v values were also associated with a lower risk of death, with HRs of 0.38 (95% CI 0.35–0.41) and 0.88 (95% CI 0.80–0.96), respectively. The hemodiafiltration cohort exhibited a lower risk of death, with an HR of 0.29 (95% CI 0.11–0.77), and this association was statistically significant with a *p*-value of 0.013, as detailed in Table 2.

**Figure 3:**
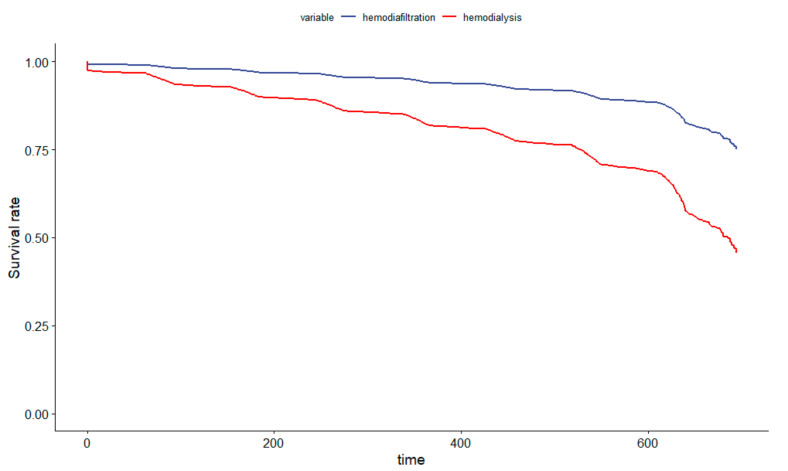
Survival-Adjusted Plot Comparing Hemodiafiltration to the Hemodialysis Cohort in Brazil.

Figure 3 displays an adjusted survival plot to compare HDF with the hemodialysis group.

### Sensitivity Analysis

We conducted a propensity score analysis employing a 2:1 optimal matching approach. This analysis retained all hemodiafiltration patients and selected the most suitable matched controls (hemodialysis patients) at a 2:1 ratio, taking into account baseline characteristics and comorbidities. The final analysis included 85 hemodiafiltration patients and 170 matched hemodialysis patients (Table 3). Subsequent to matching, all standardized mean differences for covariates were below 0.1 (with a distance of 0.0002), indicating a satisfactory level of balance.

In the Cox regression analysis following propensity score matching, the hemodiafiltration group exhibited a lower risk of death, with a hazard ratio (HR) of 0.32 (95% CI 0.11–0.91), yielding a statistically significant *p*-value of 0.032.

**Figure 4:**
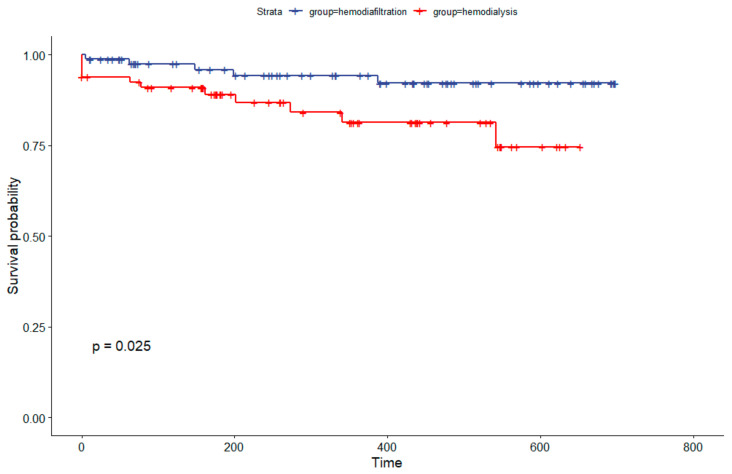
Kaplan–Meier Survival Analysis Comparing Hemodiafiltration to Hemodialysis Cohort in Brazil After Propensity Score Match.

The Kaplan–Meier analysis following propensity score adjustment demonstrated survival rates for hemodiafiltration and hemodialysis at 180 days of 96% and 89%, respectively. At the conclusion of one year, the survival rates were 94% for hemodiafiltration and 81% for hemodialysis, indicating a statistically significant difference (*p* = 0.025), as shown in Figure 4.

The calculated E-value (hazard ratio) for mortality in multivariate Cox regression was 4.70, with an upper confidence bound of 4.31.

## 4. Discussion

In this study, we compared a cohort of hemodiafiltration patients with a large cohort of hemodialysis patients using real-world data in Brazil. We concluded that hemodiafiltration was independently associated with a lower risk of all-cause mortality in a real-world scenario.

The hemodiafiltration cohort in this study exhibited characteristics similar to those of hemodialysis patients in Brazil. According to the Brazilian Hemodialysis Census of 2021, diabetes accounted for 30% of baseline kidney diseases, hypertension for 32%, and unknown etiology for 15% [14]. The type of vascular access was comparable between the two groups, with arteriovenous fistula prevalence ranging from 61% to 63%. Age, gender, and single-pool Kt/V values were similar across both hemodiafiltration and hemodialysis cohorts. We found some differences in monthly biochemical exams, favoring the hemodiafiltration cohort with superior adequacy parameters, such as higher hemoglobin levels and lower parathormone values.

The hemodiafiltration cohort achieved a session volume of 24 ± 10 L, which qualifies as high-volume convective ultrafiltration. In line with the guidelines from the European Dialysis (EUDIAL) working group, low-convective treatments fall below 15 L per session [15]. Additionally, the mean convective volume in the present study closely resembled that achieved in the CONVINCE trial, which stood at 23 L per session [9].

The theoretical advantages of hemodiafiltration over hemodialysis are associated with its capacity to effectively eliminate large molecules. High-volume hemodiafiltration (HDF) appears to be the most effective technique for the comprehensive removal of small, medium-sized, and large molecules. A noteworthy example is β2-Microglobulin, with a molecular weight of 11,800 Daltons, which cannot be efficiently eliminated by low-flux hemodialysis due to its size exceeding the membrane pore. Its clearance primarily relies on high-flux dialysis and convective processes [16]. These could be translated clinically into better control of hyperphosphatemia [8], a better erythropoietin response [17], and superior survival related to lower cardiovascular risk [18]. In the present study, we found lower values of parathormone and higher values of hemoglobin in the hemodiafiltration cohort compared to the hemodialysis group. Considering the target Kidney Disease Outcomes Quality Initiative (KDOQI) values for hemoglobin (10 g/dL) and parathormone (600 pg/mL), a higher proportion of HDF patients were in line with those thresholds.

Several observational studies have reported reduced mortality rates in patients undergoing hemodiafiltration (HDF) [19,20]. For instance, the Dialysis Outcomes and Practice Patterns Study (DOPPS) demonstrated a 35% reduction in mortality risk among patients treated with high-efficiency HDF [19]. Subsequently, randomized controlled trials (RCTs) were conducted to compare HDF with hemodialysis (HD) [21,22]. These RCTs, however, failed to establish survival advantages associated with different therapies [21,22]. The absence of statistically significant advantages for HDF in these RCTs may be attributed to factors such as limited case numbers, insufficient follow-up duration, or an inability to achieve the required minimum convective volume [23]. More recently, the CONVINCE trial provided evidence of a survival benefit for high-flux hemodiafiltration over hemodialysis in a controlled randomized trial [9].

The results of the present study demonstrate a lower mortality rate associated with hemodiafiltration compared to hemodialysis, with a hazard ratio (HR) of 0.29 (95% CI 0.11 to 0.77), adjusting for baseline characteristics, dialysis adequacy, and biochemical parameters. The results were similarly observed when employing a 2:1 optimal match propensity score, revealing a hazard ratio (HR) of 0.32 (95% CI 0.11–0.91) with a corresponding *p*-value of 0.032. Notably, the reduction in mortality observed in the present study exceeded that reported in the CONVINCE trial [9]. We observed a significant 71% reduction in mortality, in contrast to the 23% reduction in the CONVINCE trial [9]. This difference can be attributed to the fact that the CONVINCE trial compared high-flux hemodialysis with hemodiafiltration, while our current study compared standard hemodialysis with high-flux hemodiafiltration. To ensure our results were not biased and guarantee a more precise comparison between HDF and hemodialysis, we chose not to exclude incident patients (those with less than 3 months of therapy) from the HDF group.

The exceptionally low mortality rate in our hemodiafiltration cohort could be attributed to the fact that the majority of HDF patients achieved a high convective volume (24 ± 10 L per session), with 47% undergoing treatment at a frequency ranging from 5 to 6 sessions per week. Consistent with these findings, the Turkish Online Hemodiafiltration Study demonstrated a 71% reduction in cardiovascular mortality in the high-flux hemodiafiltration cohort (more than 17.4 L convective volume) compared to hemodialysis [22]. On the contrary, the mortality rate in the hemodialysis group was notably high, at 14%. However, previous studies have consistently demonstrated elevated mortality rates in the hemodialysis population in Brazil. According to the Brazilian census, the mortality rates were as follows: 2017: 19.9%; 2018: 19.5%; 2019: 18.5%; 2020: 24.5; and 2023: 22.3% [14].

In this study, we analyze real-word data to validate the results of randomized controlled trials (RCTs). RCTs, while rigorous, often have limited generalizability, do not capture long-term effects, and could miss rare adverse events. Real-world data includes diverse patient populations and provides insights into practical applications, long-term outcomes, safety concerns, and cost-effectiveness [24,25]. In this line, we confirmed the mortality reduction in hemodiafiltration over hemodialysis in a real-world scenario.

This study had some limitations. First, the cohort of hemodialysis patients was sourced from a large database associated with reimbursement within the public health system in Brazil. Due to the inherent nature of these data, certain fields were inaccessible. Specifically, we were unable to retrieve information regarding the underlying kidney disease of the patients in the hemodialysis cohort. Nevertheless, it is highly likely that the baseline kidney disease and comorbidities in the hemodialysis cohort were similar to those in our hemodiafiltration cohort, as indicated by data from the 2021 Brazilian Hemodialysis Census. We were also unable to retrieve information on erythropoietin (EPO) use and dosage, which could potentially explain the differences in hemoglobin levels between the hemodiafiltration and hemodialysis cohorts. Second, there may be variations in social characteristics between the hemodiafiltration and hemodialysis cohorts that were not considered in our analysis. We implement an integrative approach for patients undergoing hemodiafiltration, involving a multidisciplinary team comprising physicians, nurses, psychologists, nutritionists, social workers, physiotherapists, and other professionals. These services may not be readily available in the public health system. Third, the data were retrieved during the COVID-19 pandemic, and we were unable to retrieve COVID-19 data for affected patients.

Despite these limitations, the significant reduction in mortality observed in our study is unlikely to be solely attributed to an unmeasured confounding variable. The E-value we obtained, which is 4.70, suggests that a substantial degree of unmeasured confounding would be required to undermine the effect estimate [13]. A confounding factor with an HR of 4.70 exceeds what is typically associated with prevalent risk factors like diabetes or other comorbidities. Additionally, we conducted a 2:1 propensity score matching analysis using an optimal match to confirm the robustness of the analysis.

## 5. Conclusions

In conclusion, our analysis indicates that patients undergoing hemodiafiltration exhibited a lower risk of all-cause mortality when adjusted for confounding factors compared to patients on hemodialysis. These results suggest that high-flux hemodiafiltration has survival advantages over hemodialysis in a real-world scenario.

## Figures and Tables

**Table 1:** Baseline Characteristics and Outcomes of Patients Undergoing Hemodiafiltration Compared to Hemodialysis in Real-World Scenarios in Brazil.

Characteristic	Hemodiafiltration, *n* = 85	Hemodialysis, *n* = 149,372	*p*-Value
Age (years)	54 (41, 67)	58 (46, 68)	0.054
Gender			
Male	52 (61%)	88,133 (59%)	0.7
Vascular Access			0.8
central venous catheter	33 (39%)	55,931 (37%)	
arteriovenous fistula	52 (61%)	93,441 (63%)	
Single-pool Ktv	1.34 (1.09, 1.62)	1.27 (1.00, 1.61)	0.4
Hemoglobin (g/dL)	11.44 (11.02, 11.97)	10.25 (8.97, 11.42)	<0.001
Hemoglobin below 10 (g/dL)	7 (8.5%)	20,447 (41%)	<0.001
Phosphorus (mg/dL)	4.84 (4.16, 5.57)	4.87 (3.91, 5.93)	0.8
Parathyroid Hormone (pg/mL)	236 (142, 376)	327 (166, 612)	<0.001
Parathyroid Hormone above 600 (pg/mL)	7 (8.5%)	26,664 (26%)	<0.001
Albumin (g/dL)	3.79 (3.63, 3.98)	3.83 (3.10, 4.00)	0.2
Duration of Follow-up (days)	391 (168, 592)	402 (101, 920)	0.15
12-month mortality	5 (5.9%)	20,478 (14%)	0.036

Continuous variables are expressed as medians and percentiles of 25 and 75%. Categorical variables are expressed as numbers and percentages.

**Table 2:** Multivariate Cox Regression Analysis of Patients Undergoing Hemodiafiltration Compared to Hemodialysis in Real-World Scenarios in Brazil.

Characteristic	HR	95% CI	*p*-Value
Group			
Hemodialysis	—	—	
Hemodiafiltration	0.29	0.11, 0.77	0.013
Age (years)	1.03	1.02, 1.03	<0.001
Vascular Access			
central venous catheter	—	—	
arteriovenous fistula	0.38	0.35, 0.41	<0.001
Single-pool Ktv	0.88	0.79, 0.98	0.006
Hemoglobin (g/dL)	0.78	0.77, 0.80	<0.001
Phosphorus (mg/dL)	1.06	1.03, 1.09	<0.001

**Table 3:** Baseline Characteristics and Outcomes of Patients Undergoing Hemodiafiltration Compared to Hemodialysis in Real-World Scenarios in Brazil After Propensity Score Match.

Characteristic	Hemodiafiltration, *n* = 85	Hemodialysis, *n* = 170
Age (years)	54 (41, 67)	54 (40, 64)
Gender		
Male	52 (61%)	103 (61%)
Vascular Access		
central venous catheter	33 (39%)	76 (45%)
arteriovenous fistula	52 (61%)	94 (55%)
Single-pool Ktv	1.32 (1.11, 1.60)	1.27 (1.27, 1.32)
Hemoglobin (g/dL)	11.41 (10.99, 11.97)	10.80 (10.26, 11.91)
Phosphorus (mg/dL)	4.87 (4.17, 5.57)	4.87 (4.21, 4.87)
Parathyroid Hormone (pg/mL)	239 (144, 374)	253 (108, 327)
Albumin (g/dL)	3.80 (3.63, 3.97)	3.83 (3.83, 4.00)
12-Month mortality	5 (5.9%)	22 (13%)

Continuous variables are expressed as medians and percentiles of 25 and 75%. Categorical variables are expressed as numbers and percentages.

## Data Availability

The data presented in this study are available on request from the corresponding author. The data are not publicly available due to privacy considerations.
